# A Prospective Analysis of Genetic Variants Associated with Human Lifespan

**DOI:** 10.1534/g3.119.400448

**Published:** 2019-08-26

**Authors:** Kevin M. Wright, Kristin A. Rand, Amir Kermany, Keith Noto, Don Curtis, Daniel Garrigan, Dmitri Slinkov, Ilya Dorfman, Julie M. Granka, Jake Byrnes, Natalie Myres, Catherine A. Ball, J. Graham Ruby

**Affiliations:** * Calico Life Sciences LLC. 1170 Veterans Blvd, South San Francisco 94080; † Ancestry, San Francisco, California 94107, and; ‡ Ancestry, Lehi, Utah 84043

**Keywords:** GWAS, human, lifespan

## Abstract

We present a massive investigation into the genetic basis of human lifespan. Beginning with a genome-wide association (GWA) study using a de-identified snapshot of the unique *AncestryDNA* database – more than 300,000 genotyped individuals linked to pedigrees of over 400,000,000 people – we mapped six genome-wide significant loci associated with parental lifespan. We compared these results to a GWA analysis of the traditional lifespan proxy trait, age, and found only one locus, *APOE*, to be associated with both age and lifespan. By combining the *AncestryDNA* results with those of an independent UK Biobank dataset, we conducted a meta-analysis of more than 650,000 individuals and identified fifteen parental lifespan-associated loci. Beyond just those significant loci, our genome-wide set of polymorphisms accounts for up to 8% of the variance in human lifespan; this value represents a large fraction of the heritability estimated from phenotypic correlations between relatives.

A great deal of effort has been exerted to quantify the variation in human lifespan that is attributable to either genetic (Vijg and Campisi 2008) or environmental factors (Kitagawa and Hauser 1973; Crimmins and Saito 2001; Chetty *et al.* 2016). The magnitude of the genetic contribution to variation in human lifespan, *i.e.*, its heritability, can be difficult to measure, and is confounded by phenomena such as assortative mating and sociocultural inheritance; nonetheless, we have previously estimated it to be under 10% (Ruby *et al.* 2018). Beyond genetics, numerous sociological and economic factors are known to affect life expectancy at both the level of the individual (*e.g.*, income, education level, employment status, marital status) and the community (*e.g.*, income inequality, access to health care) (Braveman *et al.* 2005; Murray *et al.* 2006; Jha *et al.* 2006; Backlund *et al.* 2007). Further, race as a social construct affects many of these socioeconomic factors that influence life expectancy (C. J. L. Murray *et al.* 2006; Backlund *et al.* 2007; Chetty *et al.* 2016). Genetic population structure and race are neither equivalent nor fully-independent of one another, confounding their effects. Similarly, the intermingling of genetic with sociocultural inheritance confounds the isolation of genetic contributions to variance in lifespan.

Our goal is to extend these efforts to find specific genetic variants affecting lifespan. The standard tool for elucidating the genetic architecture of polygenic traits is the genome-wide association (GWA) study. Usually, GWA studies test whether there exists a correlation between individuals phenotyped for the trait of interest and genotyped at a panel of common SNP variants. As a phenotype, lifespan challenges this approach: genotypes are generally gathered from living persons, whereas lifespan (total elapsed time between birth and death) is a property of deceased persons. Due to this challenge, current age has been used as a lifespan proxy trait in many human-aging GWAS (Schächter *et al.* 1994; Willcox *et al.* 2008; Newman *et al.* 2010; Soerensen *et al.* 2010; Deelen *et al.* 2011; Nebel *et al.* 2011; Beekman *et al.* 2013; Soerensen *et al.* 2013; Sebastiani *et al.* 2013; Deelen *et al.* 2014; Broer *et al.* 2015; Sebastiani *et al.* 2017). Logically, alleles that reduce lifespan should become depleted in older individuals, and vice-versa for alleles that extend lifespan. However, the retrospective sampling of young and old individuals potentially captures other differences between birth cohorts, and it is unclear whether this is an appropriate proxy for lifespan GWA analyses.

An alternative approach to investigate the genetic basis of lifespan is to prospectively measure the lifespan of deceased parents from specific birth cohorts and use the genotypes of their offspring in an association mapping analysis. The imperfect sharing of genotype between a parent and child dilutes the strength of statistical associations in such a study, thereby requiring very large sample sizes to identify high-confidence variants. This method was used in analyses of the United Kingdom Biobank (UKB) to identify loci significantly associated with parental lifespan (Pilling *et al.* 2016; Joshi *et al.* 2016; McDaid *et al.* 2017; Mostafavi *et al.* 2017; Pilling *et al.* 2017; Joshi *et al.* 2017). These candidate lifespan loci have yet to be tested in an independent cohort of significant size.

We investigated genetic factors affecting lifespan in a de-identified snapshot of more than 300,000 *AncestryDNA* customers that provided prior consent to participate in research and were linked to publically visible family trees. We estimated genetic ethnicity and found it to be significantly correlated with parental lifespan. To identify genetic variants that affect human longevity, we conducted GWA mapping analyses of parental lifespan and identified five loci significantly associated with paternal lifespan and one locus associated with maternal lifespan. We estimated the phenotypic variation in lifespan attributable to a genome-wide set of variants to be quite low (between 2 and 8% for different sub-cohorts) and the genetic correlation between maternal and paternal lifespan to be quite high (at least 68%). We conducted GWA analyses of the lifespan proxy trait, age, and identified a single locus, *APOE*, associated with both traits (age and lifespan). We also observed strong genetic correlations between the two traits (43–70%). Finally, we analyzed an independent UKB dataset in a meta-analysis of more than 650,000 individuals and corroborated the association at four of the six loci identified in the *AncestryDNA* analysis. In total, this meta-analysis identified eleven paternal and four maternal lifespan-associated loci, two of which have not previously been associated with parental lifespan.

## Materials and Methods

### Set of aggregated ancestry pedigrees provides parental lifespan data

To measure parental lifespan, we interrogated a large, non-redundant set of aggregated and anonymized pedigrees, referred to as SAP. The SAP is constructed from overlapping *Ancestry* customer-generated family trees designated as “public” (Ruby *et al.* 2018). Aggregation occurred by identifying and collapsing redundant identical ancestors and stitching them together across pedigrees. The methods used to assess data quality, compare nodes between customer-generated pedigrees and aggregate this information into the SAP used in this study are described by Ruby *et al.* (2018). To protect customer privacy, SAP data were de-identified with randomly generated IDs and presenting birth and death dates as years, a lower resolution than was available in the source data.

Lifespan was measured on every record that had a year of birth and death. We considered lifespan values between 40 and 120 years to reduce early-life contributions to environmental variance and remove likely errors in reporting of birth or death dates. We did not estimate the Cox proportional hazard of mortality, as in other analyses (Joshi *et al.* 2016; Pilling *et al.* 2017), because of the prevalence of missing year of death data in the SAP. Approximately 1/3 of individuals starting in 1900 and going back multiple centuries lack a death date (Ruby *et al.* 2018, Figure 1G) even though they are assuredly deceased. Therefore, when an individual was missing year of death data we could not determine whether: 1) the individual was still alive and legitimately right-censored, or 2) the individual was deceased and the death date was never added to the genealogy.

**Figure 1:**
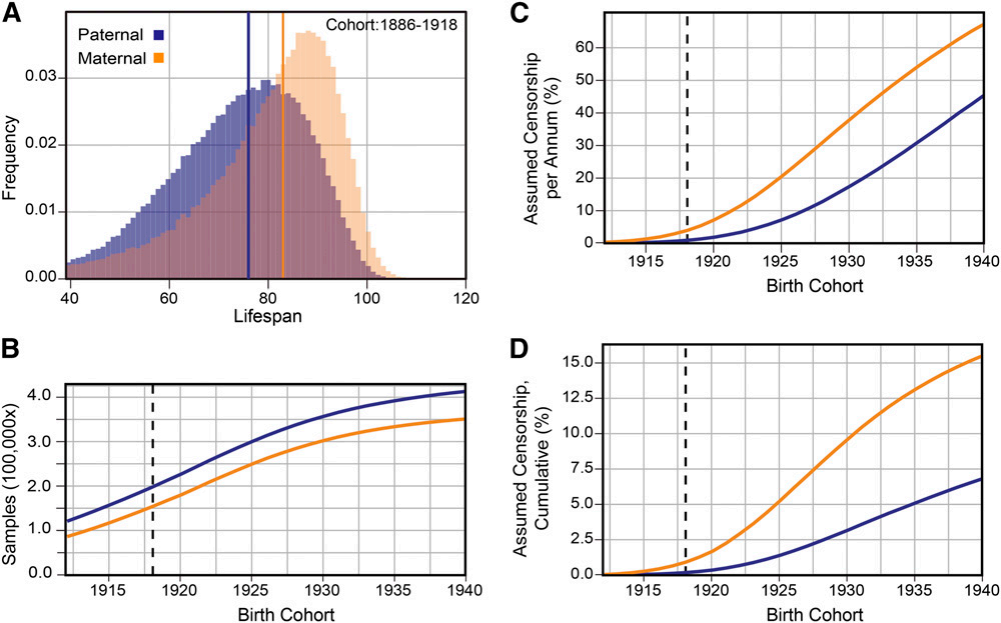
(A) Distribution of parental lifespan values from the 1886-1918 birth cohort. Median values noted with colored vertical lines. (B) Cumulative number of genotyped individuals with complete lifespan data. Birth cohort span from 1886 to year on x axis. Vertical gray line denotes the 1918 cohort. (C) Estimated number of long-lived individuals assumed to be censored as fraction of annual cohort size. (D) Estimated number of long-lived individuals assumed to be censored as a fraction of cumulative cohort size. In all figure panels, paternal individuals are denoted with blue, maternal individuals with orange, and overlapping values are dark orange.

Although we were unable to determine whether any specific individual missing year of death data were alive or dead, we estimated the fraction of individuals that were alive, and assumed to be right-censored, in the 1911-1940 birth cohorts using the distribution of lifespans from the 1886-1910 cohort as a baseline. We used the lifespan distribution from 1886-1910 birth cohort as our baseline because, in the year 2016, it contained complete lifespan data for all individuals from the 1910 birth cohort with a lifespan less than or equal to 106 years, all lifespans from the 1909 birth cohort, equal to or less than 107 years, and so on. Thus, we expect few individuals to be truly censored from this baseline.

### IRB statement regarding data use

All data required for this research project was collected previously with informed consent for purposes consistent with this research and was used for this research only after removing all personally identifying information. An IRB reviewed this research project and determined no further approval was required.

### Genotype sample collection

Data utilized for this research project was de-identified prior to its use. The laboratory extracts DNA from a sample and genotyped it using an Illumina array (details below). In order to be included in this study, customers must: 1) activate a DNA test and agree to *AncestryDNA’s* terms and conditions, privacy statement, including an explicit consent to process their DNA, 2) agree to the informed consent to research agreement, and 3) provide basic personal information - including year of birth, name, and sex. Next, the customer must have associated their DNA sample to their own user-generated pedigree and made this pedigree public to other *Ancestry* users. This analysis was based on samples submitted between May 2012 and June 2016. The database snapshot occurred on June 19, 2016 and contained 698,812 genotyped individuals linked to the SAP.

### Array based genotyping procedure

SNP variants were called by technicians at the Illumina FastTrack Microarray Services lab using the GenomeStudio platform. Genotype data were generated using an Illumina genotyping array with approximately 730,000 SNPs. During the four years of sample collection, two versions of the array were used for genotyping. All downstream analyses used the intersection of SNPs present on both versions of the chip. We included chip version as a covariate in all association mapping models.

### Estimates of genetic ethnicity and test of association with parental lifespan

We used 112,909 SNPs to estimate the genetic ethnicity of de-identified genotyped individuals who consented to research and were associated with public family trees. Genetic ethnicity is the proportion of their genome that matches a reference panel of 3000 individuals from 26 ancestral populations (described in: Ball *et al.* 2013; Han *et al.* 2017). An individual’s genetic ethnicity proportions reflect ancestral admixture events between these reference populations.

We measured whether ancestral admixture proportions were independent of each other using the Pearson correlation coefficient, *r*. We use multivariate linear regression models to test for a correlation between percent ancestral admixture and parental lifespan. Lifespan was calculated from the mothers and fathers of genotyped individuals from the 1886-1918 birth cohort. In these models, we included all ancestral populations with a minimum of 1000 individuals which were born between 1886-1918 and possessed greater than 5% admixture assignment. The 14 ancestral populations that met these requirements were: Native American, Caucasus, Great Britain, Ireland, Europe East, Scandinavia, Italy/Greece, Europe West, Iberian Peninsula, European Jewish, Finland/NW Russia, Near East, Sub-Saharan Africa and Hispanic/Latino.

### Principal component measures of population structure

In addition to genetic ethnicity, we measured population structure using a principal component analysis (PCA) of a genome-wide panel of SNPs. Given the large size of this snapshot, we estimate the first 10 principal components using the fastPCA algorithm, method: *randomized*, implemented in in the R package *SNPRelate* (Zheng *et al.* 2012). For this analysis, we used 50,000 randomly selected SNPs that were in approximate linkage equilibrium with each other (LD < 0.2). This procedure was implemented in PLINK v1.9 (Chang *et al.* 2015). We included the first 10 principal components as covariates in all association mapping models.

### IBD measurement between individuals

Given the nature of the resource, it is not surprising that a significant number of closely related individuals are represented in the snapshot. To reduce the effects of shared environmental variation due to local household environment and more closely meet the assumption of genotype independence in GWA mapping analyses, we removed individuals until no pair of samples exhibit more than 300cM identity by descent (IBD). IBD was measured with a custom algorithm, J-GERMLINE (Ball *et al.* 2016) based upon the GERMLINE algorithm (Gusev *et al.* 2009). Since shorter IBD segments are difficult to accurately identify and are less informative for estimating familial relationships, we used the ‘Timber’ algorithm to filter uninformative matches (Ball *et al.* 2016). This caused a skew in the distribution of IBD matches less than 90 cM.

### Simulation of GWA analysis of parental traits using offspring genotypes

In order to estimate the statistical power of our mapping populations, we simulated a GWA mapping analysis in which the phenotypes and genotypes were measured in offset generations. Briefly, we modeled a situation in which a single, additive allele effects our focal trait and segregates in a large, random mating population at Hardy-Weinberg equilibrium. We randomly assigned genotypes in the parental generation. Next, we calculated the parental trait value given their genotype and environmental variance, modeled with a random-normal distribution. The offspring genotype was calculated using the genotype of a parent where the trait value was known and a second parent, randomly sampled from the population. We assumed perfect Mendelian segregation of alleles between generations. Finally, we regressed the offspring’s genotype against a single parental phenotype.

Simulations were run varying the effect allele frequency (from 0.001 to 0.5) and the additive effect size of a single copy of the allele (from 0.06 to 3.8 years). We simulated mapping populations of 80,000 to 480,000 individuals. Fifty replicate simulations were run for each parameter combination. Power was measured as the number of replicate simulations which detected a significant effect (*P* value less than 5.0e-8) of the focal allele *vs.* the total number of simulations for each parameter combination. For each simulation we also calculated the allelic effect inferred from the genotypes of the parents *vs.* genotypes from the offspring.

### Parental lifespan GWA mapping analyses

We conducted quantitative GWA mapping analyses of parental lifespan using PLINK v1.9 (Chang *et al.* 2015). We conducted separate analyses for paternal and maternal lifespans. The majority of genotyped individuals were linked to two parental lifespans and we did not wish to test the same genotype against multiple phenotypes in a single GWA analysis. We further sub-divided our cohort and ran separate GWA analyses on the three broad population groups: European, Sub-Saharan African, and East Asian/Native American. For each mapping population, we identified close genetic relatives (IBD greater than 300 cM) and randomly removed one of the individuals from the pair. We examined the genetic basis of lifespan of parents born in both the 1886-1918 and 1886-1940 cohorts.

We applied the following genotype filters: minor allele frequency (MAF) threshold of 0.5%, variant missingness threshold of 20%, an individual missingness threshold of 2%, and 1% of SNPs with strongest deviation from Hardy-Weinberg Equilibrium. Additionally, we used the ‘–indep-pairwise’ function in Plink v1.9 to filter sites in which linkage disequilibrium with an adjacent site exceeded 0.9. After applying these filters, between 540,852 and 541,614 SNPs were included in any one GWA mapping analysis. We included the mitochondria and Y chromosomes in our initial analyses but found no effect (results not shown) and removed them from all subsequent analyses. The array version and first ten PCs were added as covariates to the association mapping models.

To identify lifespan-associated variants, we controlled for the genomic variance inflation factor, λ, and used a Bonferroni correction to establish a threshold of statistical significance at *P* = 9.2e-8. For each lifespan associated variant, we ensured that missingness was less than 5%. Next, we estimated the phase of each individual genotype for 5.0 megabase (Mb) regions surrounding an associated variant using the *AncestryDNA* algorithm, Underdog (Ball *et al.* 2016). We additionally imputed all cataloged variants from each 5.0 Mb region using the 1000 Genomes (1000G) phase 3 reference panel (The 1000 Genomes Project Consortium *et al.* 2015). Imputation was performed using the default parameters in minimac3 (Das *et al.* 2016).

### Genetic heritability analyses

We used LD Score Regression v1.0.0 (Finucane *et al.* 2015) to estimate the heritability, *h_g_^2^*, of lifespan using genotyping array variants that passed all quality control filters. For all heritability estimates, we used LD scores measured using the *AncestryDNA*, European population group, rather than the 1000G European (1000G,EUR) population LD score estimates included with the program. For this analysis, we included 482,066 male and female individuals; this sample contained no relatives with pairwise IBD scores greater than 300cM. We estimated the LD scores of 542,710 variants in 1 cM windows. Our analyses with the *AncestryDNA* LD scores produced *h*^*2*^_g_ intercept values close to 1.0 and ratio values lower than the 1000G,EUR LD scores, which suggested that these scores adequately captured the LD structure of the *AncestryDNA* dataset.

### GWA analysis of lifespan proxy trait: age

We conducted GWA analyses on the age of genotyped individuals from the European population group. We removed close genetic relatives (IBD greater than 300 cM) from this mapping population. Age, at a resolution of years, was calculated by subtracting 2016 from an individual’s birth year. We assumed all of these individuals were still alive, because all individuals recently provided a saliva sample in the four years since the launch of *AncestryDNA* (http://www.ancestry.com/corporate/about-ancestry/company-facts) and the time of this snapshot. In contrast to the lifespan phenotype, right-censorship of lifespan is an explicit assumption of the age trait and therefore addressed a potential confounder.

We conducted GWA analyses using both the case/control as well as quantitative trait frameworks. In the case/control analyses, the “cases” were individuals above a specified age, and the “controls” were individuals below another specified age, with a gap existing between the two age thresholds. The age threshold for the control population was set to the median age: 65 years for men and 64 years for women. We investigated two thresholds for the case population: 2.5% (male: 91 years, *N* = 5,860; female: 92 years, *N* = 6,803) and 5% (male: 86 years, *N* = 12,288; female: 87 years, *N* = 13,265). We reported results from the 5% case threshold. No additional loci were identified in the 2.5% case threshold GWA analysis (data not shown). We conducted GWA analyses for each gender separately (male *N*= 117,396, female *N*= 140,017) as well as together (*N* = 257,413) and included gender as a covariate in the association mapping model. To increase the mapping population size, we also conducted a quantitative trait GWA mapping analysis with all individuals of both genders between the ages of 40 and 120 years into a single analysis (*N*= 482,066).

### Genetic correlation analyses

We used LD Score regression (Finucane *et al.* 2015) to estimate the genetic correlation, *r_g_*, between: 1) maternal and paternal lifespan; and 2) the age of genotyped individuals and parental lifespan. To estimate the genetic correlation between traits, we constrained the *h*^*2*^_g_ intercept to 1.0 for both traits and the genetic covariance intercept to: r_p_*N_12_ / sqrt(N_1*_N_2_) where *r_p_* is the phenotypic correlation, N_1_ is the number of samples in study 1, N_2_ is the number of samples in study 2, and N_12_ is the sample overlap (Bulik-Sullivan *et al.* 2015).

For our estimate of the genetic correlation between age and lifespan, we required all samples to have both age and parental lifespan measurements within each gender specific comparison. Second, we aimed to minimize biases associated with younger individuals having a higher rate of parental lifespan censorship compared to older individuals, by restricting the birth cohort of genotyped individuals included in the analysis to be between the years 1916 and 1956. This resulted in an age distribution of genotyped individuals to be between 60 and 100 years. Parental lifespan was examined for the 1886-1940 birth cohort, and the distribution of lifespans examined were between 40 and 120 years.

### Cohort specific effects of lifespan associated loci

To investigate variation in the association of SNP variants and lifespan between birth cohorts, we ran separate association mapping analyses for 4 – 8 year slices spanning the 1886-1940 cohort. The cohorts interrogated were: 1886-1894, 1894-1902, 1902-1908, 1908-1912, 1912-1916, 1916-1920, 1920-1924, 1924-1928, 1928-1932, 1932-1936, 1935-1940. The number of individuals in the narrowly defined birth cohort slices range from 10722 – 23211 for paternal lifespan and 6764 – 22469 for maternal lifespan. We also measured the change in MAF between cohorts for variants at each lifespan associated locus. For *APOE*, the one locus that exhibited a change in MAF between cohorts, we also measured the allele frequency in samples binned by lifespan value.

### Comparison of AncestryDNA and UK Biobank parental lifespan analyses

We compared the *AncestryDNA* GWA of parental lifespan from the broadly defined birth cohort (1886-1940) to the largest UKB association analysis of parental lifespan published to date: Pilling *et al.* (2017). The focal trait in the UKB analysis was attained parental age, which was calculated using the Cox proportional hazard model and includes our focal trait, complete lifespan, as well as the current age of parents which were not deceased (Pilling *et al.* 2017). We downloaded GWA summary statistics from the UKB analysis of attained parents age from: https://figshare.com/articles/Plling_et_al_2017_UKB_parents_attained_age_GWAS/5439382/2.

We conducted a meta-analysis of more than 658,000 individuals using the sample size based approach implemented in *METAL* (Willer, Li, and Abecasis 2010). We included SNP variants that were common to both studies, possessed a cohort-specific MAF of greater than or equal to 0.5% and had matching alleles. With these requirements, we interrogated 458,525 and 458,865 variants in our meta-analyses of maternal and paternal lifespan. This sub-set of SNPs meant that we were only able to include variants that were found to be significant in the UKB analysis at 10 of 14 paternal attained-age loci and four of six maternal attained-age loci. We controlled for the genomic inflation factor in each dataset within METAL and established a *P* value of 5.0e-8 as our significance threshold.

We measured the genetic correlation between *AncestryDNA* and UKB GWA analyses using LD Score Regression (Finucane *et al.* 2015). We ran analyses with LD scores from both the *AncestryDNA* European population group and the 1000G,EUR population. We reported results from the *AncestryDNA* LD scores analysis because the *h^2^* ratio values were lower and the *h*^*2*^_g_ intercept values were closer to 1.0, suggesting that these LD scores are a better match than the 1000G,EUR to the genetic structure of the UKB mapping population (Finucane *et al.* 2015). In contrast to the LD score analyses of solely the *AncestryDNA* GWA results, we did not constrain the *h*^*2*^_g_ or genetic covariance intercepts in these analyses because we could not estimate the phenotypic correlation and amount of sample overlap between the two datasets. The GWA meta-analyses and LD score analyses used *z*-score transformed effect sizes from the *AncestryDNA* and UKB GWA mapping results. In order to make the effect sizes consistent with the values reported in the UKB analysis, we used effect sizes from the *AncestryDNA* analysis calculated with offspring genotypes -rather than effects sizes re-scaled to reflect allelic dosage in the parental generation.

### Privacy considerations & data availability

Supplemental Figures and Tables, with their Legends, are provided as a single PDF document: Supplemental Tables & Figures. In addition, Supplemental Tables 8 and 9 are provided as Excel documents and contain SNP-by-SNP summary statistics from each presented GWAS for representative SNPs with genomic control adjusted *P* values less than 3.0e-3 in the *AncestryDNA* cohorts (Supplemental Table 8) or 2.5e-3 in the *AncestryDNA*-UKB meta analyses (Supplemental Table 9). 

The growing literature and commentary on genomic privacy guided our decision to release summary statistics for only a subset of SNPs in this instance. The power to use an individual’s genome-wide variant data to infer whether that individual was present in or absent in a GWA cohort using association mapping summary statistics has not yet been exhaustively explored, but has nonetheless been considered from both theoretical (Visscher and Hill 2009; Sankararaman *et al.* 2009) and practical perspectives (Homer *et al.* 2008; Im *et al.* 2012; Gymrek *et al.* 2013; Cai *et al.* 2015). These studies consistently identify the ratio of reported SNP statistics to cohort size as the key factor governing identifiability. This relationship is typically described as *m* independent SNP markers, *n* individuals, with the power to identify individuals is proportional to sqrt(*m*/*n*) (Equation 1; Sankararaman *et al.* 2009). As a result, we err on the side of caution in protecting *Ancestry* customers’ privacy.

In our Supplemental Tables 8 and 9, we provide abbreviated summary statistics for seven GWA mapping analyses (for a total of 10,171 unique SNPs). Our basis for this was inclusion of all SNPs with *P* values less than 3.0e-3 for the five *AncestryDNA* specific analyses and 2.5e-3 for the two *AncestryDNA*-UKB meta analyses. This standard ensured that all significant and near-significant results were reported, and also that all other SNPs were known by the reader to be non-significant. For our smallest-cohort analysis (maternal lifespan for the 1886-1918 birth cohort: 133,203 individuals), we report 1,471 snps, which is an *m*/*n* ratio of 0.011, substantially below the ratios analyzed in the privacy literature noted above and therefore presumably safe. In contrast, the community standard of GWAS data disclosure – provision of summary statistics for all 540,852 analyzed variants – would produce an *m*/*n* ratio greater than 4.0. Re-identification power for such a high ratio is plotted by Sankararaman *et al.* (2009; Figure 1; *m*/*n* = 1.0, plotted for *m* = 1,000). While low, the risk of re-identification in this scenario is not negligible.

Our report of summary statistics across seven GWA analyses increases the risk of re-identification. The work of Im *et al.* (2012) clearly identifies multi-phenotype GWAS as adding tremendous power to re-identification (presented in their Figure 6). Such added power derives from the ability to regress the same SNPs across multiple phenotypes; in contrast, our presentation of summary statistics for the most-significant SNPs for each analysis resulted in largely non-overlapping sets of SNPs, thereby negating the additional re-identification power that multiple GWAS provide. Had we reported summary statistics across all SNPs, then the SNPs would have been totally consistent between phenotypes. The additional power gained from the multiple phenotypes – together with the added power provided by the larger SNP set – would have profoundly facilitated re-identification, far beyond what the increased *m*/*n* ratio would imply by itself.

The GWAS literature from another commercial entity with legal obligations to the members of its study population consistently reports the 10,000 most-significant SNPs (Jansen *et al.* 2019; Shaffer *et al.* 2017; Hu *et al.* 2016; Jones *et al*. 2017; Aberg *et al.* 2018; Huusko *et al.* 2018; Lee *et al.* 2018; Jones *et al.* 2019). For our complete study, 10,171 unique SNPs provided a level of disclosure that permits scientific scrutiny while comfortably maintaining the anonymity of our users.

Ancestry’s highest priority is protecting our customers’ privacy and being good stewards of their data; that starts with the basic belief that customers should always maintain ownership and control over their own data. We have provided access to the significant findings that would allow for a level of replication in the supplemental files. We collaborate with scientific researchers on a case-by-case basis to advance science in specific areas while using research methodologies that protect consumers when appropriate. Examples to be considered in these requests include but are not limited to:

1. Technical and scientific feasibility
2. Level of detail required from the underlying data (including its sensitivity)
3. Novelty/potential impact of the proposed work
4. Academic institution (in good standing and acting in good faith while implementing appropriate technical, administrative, and physical measures to protect the privacy of the participants and the security of the data)
5. Amount of resource allocation for *Ancestry* required to support the collaboration

Correspondence and requests should be addressed to Catherine Ball (cball@ancestry.com). Supplemental material available at Figshare: https://doi.org/10.25387/g3.8479022.

## Results

### The Ancestry pedigree facilitated GWA mapping analysis of human lifespan

To measure parental lifespan, we interrogated a large, non-redundant set of aggregated and anonymized pedigrees, referred to as SAP, generated by stitching together overlapping customer-generated family trees designated as “public” (Ruby *et al.* 2018). The June 2016 snapshot of *AncestryDNA* customers contained more than 680,000 individuals who provided prior consent to research, were linked to the SAP and had at least one parent with complete lifespan information, *i.e.*, recorded year of birth and death. The majority the parents with lifespan information (76.0% of fathers and 77.7% of mothers) were born in the 1900-1930 cohort. For this cohort, nearly 80% of these individuals were born in the United States, ∼10% were born in Europe and the remaining ∼10% born outside of the US and Europe (Ruby *et al.* 2018, Figure 1I). We considered lifespan values between 40 and 120 years to reduce early-life contributions to environmental variance and remove potential errors in the recording of birth or death dates (Figure 1A).

To maximize sample size and power in the association mapping analysis, we sought to include individuals from a wide range of birth cohorts (Figure 1B). However, the addition of younger birth cohorts increased the systematic removal of long-lived, right-censored individuals, because they lack a year of death. We estimated the fraction of individuals that were assumed to be censored (*i.e.*, were missing a year of death and were presumably alive in the 1911-1940 birth cohorts) using the lifespan distribution from 1886-1910 birth cohort as a baseline (Figure 1C, D). We found the 1886-1918 cohort possessed a large sample size with minimal assumed censorship: for fathers, *N* = 208,707, assumed censorship = 0.3%; for mothers, *N*= 162,659, assumed censorship = 1.3% (Figure 1B, D). Median paternal lifespan ranged from 75 to 77 years when comparing the 1886-1894 cohort (*N* = 10,721) to the 1916-1918 cohort (*N* = 20,858; Figure S1A), whereas maternal lifespan ranged from 81-83 years across the same timespan (*N*= 6,764 for 1886-1894; *N* = 19,239 for 1916-1918; Figure S1B). A wider range of birth cohorts, 1886-1940, increased the number of parents with lifespan information (fathers: 423,817 and mothers: 359,719, Figure 1B) and increased the fraction of missing long-lived individuals, estimated to be 6.8% for fathers and 15.5% for mothers (Figure 1D).

Given the constraint of a single-generation offset between measures of phenotype and genotype, we estimated our power to detect genetic associations of various effect sizes in datasets of this scale. The power to identify an allele with an additive effect size of 0.75 years at 20% minor allele frequency in the population would be 0.90 in a cohort of 170,000 individuals and nearly 1.0 in cohorts exceeding 230,000 individuals (Figure S2). We concluded that for our snapshot, there was sufficient power to identify a genetic variant at moderate allele frequency with an effect size of 0.75 years or greater.

### Genetic measures of ethnicity were significantly associated with lifespan

A non-random distribution of lifespans with respect to the underlying genetic structure in the mapping population can cause false positives in a GWA mapping. Previous research demonstrates the association between race, defined as a socially constructed categorical variable maintained by dominant social groups (Mills 1997; Haslanger 2008), and life expectancy (C. J. L. Murray *et al.* 2006; Backlund *et al.* 2007; Chetty *et al.* 2016). We sought to determine whether there was a similar association in this snapshot, however we have no knowledge of individuals’ race, thus we instead estimated their genetic ethnicity and compared this to the lifespan of their parents.

**Figure 2:**
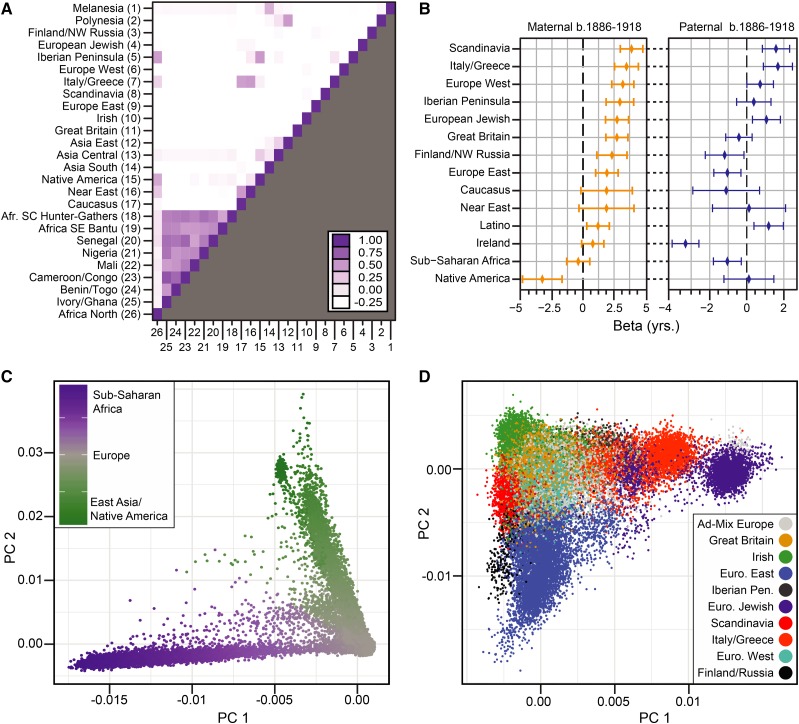
(A) Pairwise correlation between ancestral admixture proportions of genotyped individuals from analysis of paternal lifespan, 1886-1918 birth cohort. (B) Linear regression coefficient (+/−*SE*) for admixture population and paternal or maternal lifespan. The dashed line at zero represents the mean lifespan for all individuals in the analysis. (C) Population structure, measured with principal component analysis, of individuals from analysis of paternal lifespan, 1886-1918 birth cohort. Each circle is a single individual, colored according to percent composition of Sub-Saharan Africa and Native American / East Asian admixture populations. (D) Population structure within individuals of European descent, after filtering Sub-Saharan Africa, Native American/East Asian groups. Colors depict individuals with majority ethnicity assignment in one of nine European populations.

We estimated the genetic ethnicity as the proportion of the genome that is assigned to a reference panel of 26 ancestral populations for de-identified genotyped individuals who have previously consented to research and were associated with public family trees (described in: Ball *et al.* 2013; Han *et al.* 2017). We found that genetic ethnicity proportions, which represent ancestral admixture events between these reference populations, were generally uncorrelated with each other in this snapshot (Figure 2A), except in the case of the eight Sub-Saharan African ethnicities (*r* > 0.38), which we combined into a single Sub-Saharan African group; and the Native American and Iberian ancestral admixture (*r* = 0.44), which, for individuals exhibiting evidence of admixture (Han *et al.* 2017), were combined into a single Latino group.

Using multivariate, linear regression models we found that ancestral admixture proportions were significantly associated with both maternal and paternal lifespan (*P* < 2e-16) although the magnitude of the correlations were not large (maternal lifespan *r* = 0.047, paternal lifespan *r* = 0.056). We found significant, positive correlations with maternal lifespan and genetic ethnicity assignment to the following populations: Europe West, European Jewish, Scandinavia, Italy/Greece, and Great Britain (Figure 2B, Table S1). Conversely, there was a negative correlation between maternal lifespan and ancestral admixture with the Native American population, although this was not significant after multiple test correction (Table S1). Paternal lifespan was positively correlated with ancestral admixture with Scandinavian and Italian/Greek populations, although this was not significant after multiple test correction (Figure 2B, Table S2). Ancestral admixture with a single population, Ireland, exhibited a significant, negative correlation with paternal lifespan (Table S2). Given the relationship between race and life-expectancy (C. Murray *et al.* 1998; Backlund *et al.* 2007; Chetty *et al.* 2016), we inferred that non-genetic, social factors contributed to the correlation between lifespan and genetic ethnicity.

We attempted to control for these factors in our association analyses by calculating genetic differentiation using principal component analysis (PCA) and splitting this snapshot of de-identified genotyped individuals who provided prior consent to research and were associated with public family trees into one of three broad population groups: Sub-Saharan African, Native American/East Asian or European (Figure 2C). We applied a conservative minimum threshold of 5% ancestral admixture assignment inclusion in the Sub-Saharan African and Native American/East Asian groups; these samples comprised a small proportion of the snapshot. All remaining individuals were placed into the European population group. Next, we re-calculated genetic PCs for each group and used these as covariates in the association mapping analyses. GWA analyses focused on the European population group (Figure 2D) because it contained the majority of samples.

### GWA mapping revealed three loci to be significantly associated with parental lifespan in the 1886-1918 birth cohort

We first investigated the genetic basis of maternal and paternal lifespan in the 1886-1918 birth cohort. This mapping population was of primarily European descent and contained no close genetic relatives (IBD threshold 300 cM; Figure S3). The number of individuals in maternal and paternal analyses was 133,203 and 167,179, respectively. After quality control filtering of array genotyping variants, the minimum number of variants tested in each analysis was 540,852 (Table S3).

**Figure 3:**
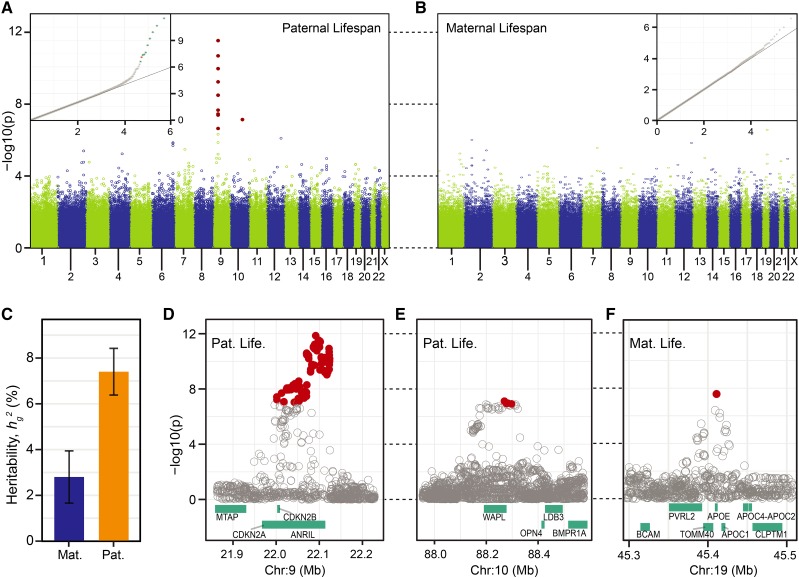
Manhattan plot of GWA results of (A) paternal and (B) maternal lifespan, cohort 1886-1918. Each circle is a single genotyped SNP, solid red circles denote SNPs with Bonferroni corrected *P* values less than 0.05. Inset: Q-Q plot with expected (x-axis) *vs.* observed (y-axis) –log10 *P* values. Non-gray points denote SNPs with Bonferroni corrected *P* values less than 0.05. Association analysis of candidate loci with imputed data: (C) Heritability of lifespan, estimate (+/−*SE*) with genome-wide panel of SNPs. (D) Chrm9: *ANRIL*, paternal lifespan (E) Chrm10: *WAPL*, paternal lifespan (F) Chrm19: *APOE*, maternal lifespan. Solid red circle are SNPs with Bonferroni corrected *P* values less than 0.05. Cyan bars: hg19 gene models.

We found genetic variants at two loci associated with paternal lifespan (Figure 3A) and no variants associated with maternal lifespan (Figure 3B) in the European population group at a Bonferroni-corrected *P* value less than 0.05, which corresponded to observed *P* value less than 9.24e-8 (Table 1). The genotyping chip version covariate was not significant in these analyses (Table S4; Figure S4). These variants remained significant after controlling for the genomic variance inflation factors: λ_Paternal_ = 1.084, λ_Maternal_ = 1.030 (Table S3). We inferred that most of this inflation was due to polygenicity, rather than unaccounted population structure, because the LD score regression intercepts were nearly 1.0 and the mean LD score regression (LDSC) χ^2^ estimates were similar to the genomic variance inflation estimates: 1.085 and 1.033 for paternal and maternal lifespan, respectively (Table S3; Finucane *et al.*, 2015).

**Table 1:** Candidate paternal and maternal lifespan loci. For each locus, we list the lead genotyped variants, and for *SRRM3* and *APOE* we also list the lead imputed variant, indicated with SNP ID prefix: bp. The additive effect of each allele at the candidate locus is given in years, scaled to the expected allelic dosage in the parental generation. *h*^2^_snp_ is the fraction of variance in lifespan explained per snp. The statistical significance of the association at each candidate variant is given as an uncorrected (*P*_UC_) value and genomic control corrected (*P*_GC_) value. Significance of *P*_GC_ after Bonferroni multiple test correction: *** *P*_Bonf_ < 0.001; * *P*_Bonf_ < 0.05; - *P*_Bonf_ > 0.05

Lifespan	Locus	RSID	Chr	Effect Allele	Effect Allele Freq.	1886 - 1918						1886 - 1940					
						Effect (years)	*SE* (years)	*h*^*2*^_snp_ (%)	*P*_UC_	*P*_GC_		Effect (years)	*SE* (years)	*h*^*2*^_snp_ (%)	*P*_UC_	*P*_GC_	
Paternal	*ANRIL*	rs1333042	9	A	0.49	0.65	0.09	0.063	4.01e-13	3.21e-12	***	0.51	0.07	0.039	7.00e-15	8.13e-13	***
	*WAPL*	rs10887623	10	G	0.10	−0.84	0.15	0.037	2.26e-08	7.89e-08	*	−0.51	0.11	0.014	3.36e-06	1.92e-05	—
	*LPA*	rs9457925	6	A	0.02	−1.66	0.33	0.030	5.49e-07	1.51e-06	—	−1.71	0.25	0.031	4.14e-12	1.83e-10	***
	*SRRM3*	rs17685	7	A	0.28	0.38	0.10	0.018	1.13e-04	2.08e-04	—	0.40	0.07	0.019	5.41e-08	5.73e-07	—
		bp75763624	7	A	0.32							0.42	0.07	0.023	2.64e-09	4.39e-08	*
	*CHRNA3/5*	rs931794	15	G	0.35	−0.34	0.09	0.017	1.92e-04	3.40e-04	—	−0.41	0.07	0.023	3.19e-09	5.17e-08	*
											—						
Maternal	*APOE*	rs4420638	19	G	0.18	−0.60	0.13	0.033	4.83e-06	6.60e-06	—	−0.49	0.09	0.022	5.94e-08	2.76e-07	—
		bp45411941	19	C	0.14	−0.83	0.15	0.048	1.86e-08	2.96e-08	*	−0.6	0.1	0.026	2.53e-09	1.61e-08	*

To estimate the additive effect of variants on lifespan, we doubled the values measured in our association mapping analyses – which used offspring genotypes – because the observed allelic dose in the genotyped offspring is half of the expected allelic dose in the parents (Figure S5; Joshi *et al.*, 2016). All effect sizes reported for the *AncestryDNA* specific analyses are scaled to the allelic dose in the parental generation (Table 1).

We estimated the fraction of phenotypic variance in parental lifespan explained by genotyping array variants, *h_g_^2^*, to be 7.38% (*SE* = 1.02%; N_snps_ = 390,234) and 2.78% (*SE* = 1.14%; N_snps_ = 385,494) for paternal and maternal lifespan, respectively, using LD score regression (Finucane *et al.* 2015) (Figure 3C). As with our variant effect size estimates, these *h_g_^2^* values have been re-scaled to the allelic dose in the parental generation (Figure S5; Joshi *et al.*, 2016).

Next, we examined whether the non-normal distribution of lifespan values (Figure 1A) contributed to genomic variance inflation with a permutation analysis of lifespan values from this dataset. We found that the distribution of λ values was centered at 1.00, and did not overlap the observed λ values (Figure S6). We also considered whether the increase in median lifespan between the years of 1886 and 1918 (Figure S1) may have altered these results. Re-running these analyses with an additional median lifespan per cohort covariate did not significantly change the distribution of *P* values across the genome (Figure S7), nor at the lifespan associated variants (Table S5).

To define each region of association more precisely, we imputed a 5.0 Mb block of sequence using the 1000G, phase 3 reference panel. The chromosome 9 locus, which exhibited the strongest association with paternal lifespan, included two annotated protein-coding genes (*CDKN2A/B*) and one annotated long non-coding RNA (lncRNA; *ANRIL*). Hereafter, we refer to this locus as *ANRIL* because the lead variants, defined by the strength of the statistical association, were in the lncRNA, rather than either of the two protein-coding genes (Figure 3D). The second locus associated with paternal lifespan included a single annotated, protein-coding gene: *WAPL* (Figure 3E), and, to our knowledge, has not been previously associated with any life-shortening diseases or lifespan. Although no variants achieved genome-wide significance in our analysis of maternal lifespan significant, the SNP with the lowest *P* value was located near *APOE*, a locus previously associated with survivorship to advanced age (Deelen *et al.* 2011; Nebel *et al.* 2011; Sebastiani *et al.* 2017) and parental lifespan (Joshi *et al.* 2016; Pilling *et al.* 2017). We imputed SNPs at this locus and identified a single variant which was significantly associated with maternal lifespan (Table 1; Figure 3F). This association was gender-specific: no imputed variants at the *APOE* locus were significantly associated with paternal lifespan (Figure S8A). In summary, we identified a total of three loci associated with parental lifespan in this narrowly defined birth cohort.

We found no variants that were significantly associated with maternal or paternal lifespan in the Sub-Saharan African or Native American/East Asian population groups (Figure S9A-D). With fewer than 10,000 individuals, these analyses were underpowered and this negative result was expected (Figure S2).

### Increased GWA mapping power in an expanded birth cohort identified one maternal and four paternal lifespan associated loci

To increase the power of our association mapping analysis, at the cost of increased censorship of long-lived individuals, we expanded the analysis to the 1886-1940 birth cohort. The European population group from this birth cohort contained, after filtering closely related individuals, 270,548 and 309,383 samples with maternal and paternal lifespans (Table S3).

**Figure 4:**
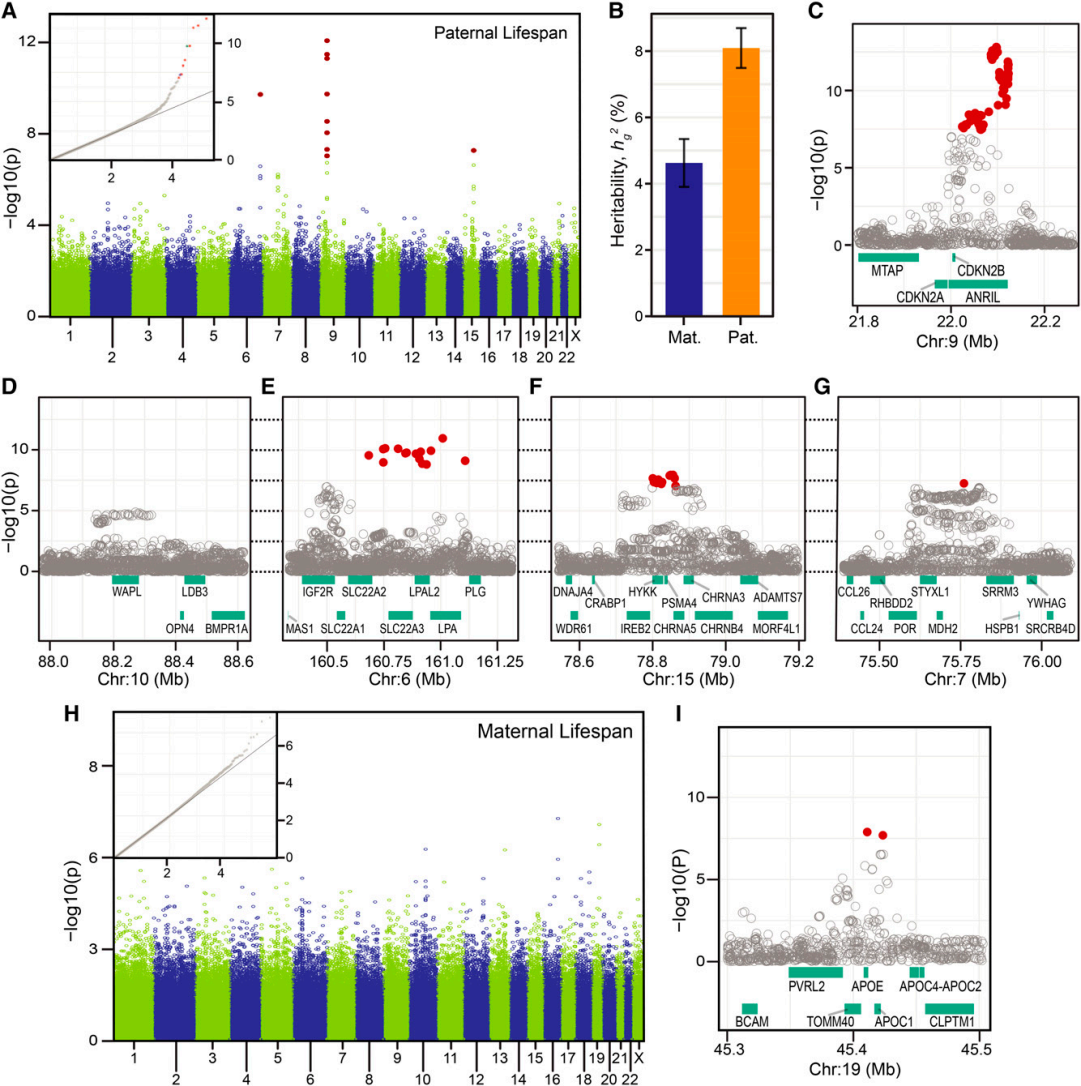
Manhattan plot of association test statistics from analyses of (A) paternal and (H) maternal lifespan, cohort 1886-1940. (B) Heritability of lifespan, estimate (+/−*SE*) with genotyped variants. Association analyses with imputed genotypes at candidate loci: (C) *ANRIL*, (D) *WAPL*, (E) *LPA*, (F) *CHRNA3/5*, (G) *SRRM3*, (I) *APOE*. Annotation details are the same as in Figure 3.

In the expanded cohort, variants at four loci (*ANRIL*, *LPA*, *CHRNA3/5*, and *SRRM3*; Table 1; Figure 4A) were significantly associated with paternal lifespan after controlling for genomic variance inflation (λ_Paternal_ =1.182; Table S3). Similar to the narrowly defined cohort, we interpreted most of this inflation was due to polygenicity, LDSCχ^2^ = 1.161 (Table S3), and *h_g_^2^* was 8.08%, *SE* = 0.60% (Figure 4B). *ANRIL* was the one locus significantly associated with lifespan in both the narrow and expanded birth cohorts (Figure 4C). In contrast, *WAPL* did not achieve genome-wide significance in the broader birth cohort (Figure 4D). We identified significantly associated variants at two loci, *LPA* and *CHRNA3/5* (Figure 4E,F), that were previously associated with paternal lifespan (Joshi *et al.* 2016; Pilling *et al.* 2017). The fourth locus, *SRRM3* (Figure 4G), was defined by a single imputed SNP (genotyping array variants were near the threshold of significance; Table 1) which, to our knowledge has not been previously associated with any life-shortening diseases or lifespan.

As with the narrowly defined birth cohort, our analysis of the expanded birth cohort identified a single locus, *APOE*, which was significantly associated with maternal lifespan (Table 1). After controlling for variance inflation, λ_Maternal_ = 1.113, none of the genotyped variants were significant (Figure 4H); however, imputed SNPs at *APOE* achieved genome-wide significance (Figure 4I). We estimated the LDSCχ^2^ to be 1.097 (Table S3) and heritability of maternal lifespan to be 4.62%, *SE* =0.72% (Figure 4B). Consistent with our findings from the narrowly defined birth cohort, *APOE* was not associated with paternal lifespan in the broadly defined cohort (Figure S8B).

We found no variants that were significantly associated with maternal or paternal lifespan in the expanded birth cohort analyses of the Sub-Saharan African or Native American/East Asian population groups (Figure S9E-H). With fewer than 20,000 individuals, these analyses were underpowered and this negative result was expected (Figure S2).

### Age was strongly associated with the APOE locus

We next sought to compare the lifespan GWA results to the commonly used proxy trait: age. We evaluated age in both the quantitative trait framework, including all individuals in a cohort (Figure S10A), and in the routinely employed case/control framework (*e.g.*, Deelen *et al.* 2011; Deelen *et al.* 2014; Broer *et al.* 2015), wherein the ‘cases’ were individuals above a specific age (*e.g.*, 5% tail: 86 years for males and 87 years for females) and the ‘controls’ were individuals below a certain age (*e.g.*, median age: 65 years for males and 64 years for females). The GWA mapping population included the European population group; individuals with close relatives in this population (IBD threshold of 300cM) were removed. In agreement with many previous studies, we identified genome-wide significant SNPs at the *APOE* locus in analyses of males, females, and combining samples from both genders (Table S6; Figure S10B-F). Additionally, we identified a SNP at the *MAP2K6* gene, which narrowly achieved genome wide significance in the case/control analysis of female age (Figure S10C).

### Genetic correlations between paternal lifespan and age

We measured the genetic correlation, *r_g_*, between the maternal and paternal lifespan and between age and lifespan using LD Score regression (Finucane *et al.* 2015; Bulik-Sullivan *et al.* 2015). We estimated the genetic correlation between maternal and paternal lifespan to be quite high: *r_g_* = 0.65 (*SE* = 0.09) for the 1886-1918 cohort and *r_g_* = 0.94 (*SE* = 0.04) in the 1886-1940 cohort (Table 2). The high genetic correlations suggest that the majority of significant allelic effects on lifespan will be similar in magnitude and direction between men and women, with a minority of alleles effecting lifespan in a gender-specific manner.

**Table 2:** Phenotypic and genetic correlations for maternal *vs.* paternal lifespan and age *vs.* lifespan. *N*- Overlap is the number of samples which have phenotypes measured for both traits. *N* - SNPs is the number of variants used in LD Score regression analysis. Phenotypic variance (P_VAR_), the slope of the correlation (beta), and the Pearson correlation coefficient (*r*_p_). Genetic heritability (*h*^2^_g_) and genetic correlation (*r*_g_)

Traits			Phenotypic Correlation			Genetic Correlation			
Lifespan	Lifespan	*N* - Overlap	P_VAR_ Mat.	P_VAR_ Pat.	*r*_p_	*N* - SNPs	%*h*^2^_g_ Maternal (*SE*)	%*h*^2^_g_ Paternal (*SE*)	*r*_g_ (*SE*)
Maternal_1918	Paternal_1918	112498	161.53	166.20	0.068	385494	3.66 (0.64)	7.54 (0.58)	0.651 (0.090)
Maternal_1940	Paternal_1940	219514	153.42	160.27	0.095	386724	5.34 (0.36)	7.62 (0.32)	0.937 (0.041)
**Age**	**Lifespan**	***N* - Overlap**	**P_VAR_ Age**	**P_VAR_ Lifespan**	***r*_p_**	***N* - SNPs**	**%*h*^2^_g_ Age (*SE*)**	**%*h*^2^_g_ Lifespan (*SE*)**	***r*_g_ (*SE*)**
Male	Maternal	90496	62.03	153.94	0.095	385974	3.04 (0.42)	4.78 (0.92)	0.700 (0.127)
Male	Paternal	98313	59.88	162.09	0.000	386086	2.68 (0.37)	7.04 (0.86)	0.432 (0.097)
Female	Maternal	109571	66.67	153.84	0.098	386209	3.69 (0.44)	4.88 (0.76)	0.693 (0.094)
Female	Paternal	115806	59.86	161.66	0.004	386293	2.61 (0.40)	7.54 (0.80)	0.527 (0.094)

Next, we estimated within and between gender genetic correlations between age and parental lifespan. Similar to the parental lifespan analysis, the genetic correlations between these traits were high: maternal lifespan compared to female and male age was 0.69 (*SE* = 0.09) and 0.70 (*SE* = 0.13); paternal lifespan compared to male and female age was 0.43 (*SE* = 0.10) and 0.53 (*SE* = 0.09) (Table 2). In summary: *h_g_^2^* for each trait was quite low (between 2 and 8% for different sub-cohorts), but the variants that underlie maternal *vs.* paternal lifespan or parental lifespan *vs.* age had substantially correlated effects.

### Effects of WAPL and APOE on parental lifespan varied across birth cohorts

**Figure 5:**
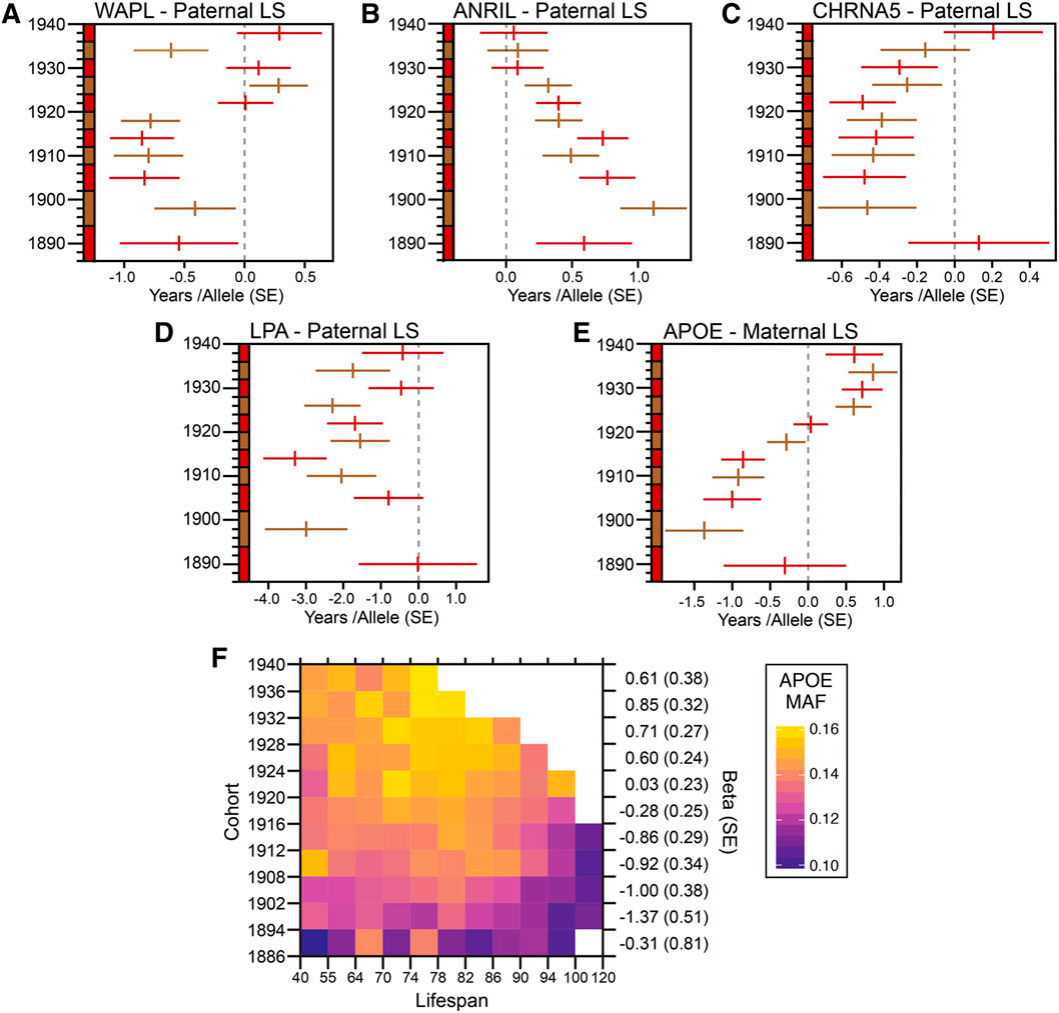
(A-E) Forest plots of allelic effect in years (+/−*SE*) of lead SNP at each candidate locus in 1886-1940 birth cohorts. Alternating red and brown lines mark successive cohorts. (F) Heat map of *APOE* minor allele frequency in the 1886-1940 birth cohorts, binned into lifespan phenotype classes spanning 40 – 120 years. White squares are missing data- birth cohort, phenotype combinations less than 500 individuals.

We investigated whether lifespan determining variants exhibited birth-cohort-specific effects because of the inconsistent *WAPL* results between the 1886-1918 (Figure 3D) and 1886-1940 (Figure 4D) cohorts: loss of significance despite increased sample size. We found that variants at *WAPL* had a mean effect of 0.70 (*SE* = 0.07) years in the 1886-1920 cohorts, and in all later cohorts, save 1928-1932, it was not significantly different from zero (Figure 5A). For comparison, *ANRIL*, *CHRNA3/5*, and *LPA* had significantly affected lifespan through 1928 (Figure 5B-D), after which we observed a decrease in mean effect size, likely due to increased censorship in the younger cohorts (assumed to be 18.5–40.9% for men in 1928-1940 cohorts; Figure 1D). In contrast, the youngest cohort in which *WAPL* had no significant effect, 1920-1924, had an assumed censorship of only 4.1% (Figure 1D). The shift in effect size could not be attributed to variation in the MAF – the standard deviation of MAF estimates, normalized to the mean, was 1.4%.

Next, we examined birth-cohort-specific effects at *APOE*, which exhibited significant negative effects on lifespan in the 1894-1920 birth cohorts and consistently positive effects in birth cohorts after 1923 (Figure 5E). Measurement of the frequency of the *APOE* allele as a function of both phenotypic class and birth cohort (Figure 5F) revealed that: 1) the MAF increased from 11.38% in older birth cohorts to 15.15% in younger cohorts; and 2) the increase was not consistent across phenotypic classes, but was most pronounced for intermediate lifespan values (74-86 years). Thus, the *APOE* allele was most strongly associated with intermediate lifespans, suggesting it may be beneficial early in life and detrimental later in life (Figure 5F). The apparent positive effect of the *APOE* allele on lifespan in cohorts after 1923 (displayed as an increase in MAF at intermediate lifespan values, 74-86 years, relative to shorter lifespan values, 40-74 years in Figure 5F) was actually due to systematic censorship of longer lifespans in younger cohorts (shown as empty squares in top right corner of Figure 5F). In summary: the minor-frequency *APOE* allele was associated with intermediate lifespan values, and this association was stronger in younger cohorts.

### GWA meta-analysis of parental lifespan with UKB dataset identified 15 lifespan associated variants

The only GWA studies of parental lifespan comparable to this analysis are derived from the UKB (Pilling *et al.* 2016; Joshi *et al.* 2016; McDaid *et al.* 2017; Joshi *et al.* 2017; Mostafavi *et al.* 2017; Pilling *et al.* 2017). Of these studies, the analysis with the largest sample size, *N* = 389,166 (Pilling *et al.* 2017) identified six and fourteen loci associated with maternal and paternal attained-age, respectively. The trait investigated in that analysis is hazard of survival, a derived variable that is inclusive of both lifespan and age. We estimated the same-gender genetic correlation between the lifespan and attained-age across the two analyses and found that these values were close to 1.0 (paternal *r_g_* = 0.98, *SE* = 0.06; maternal *r_g_* = 1.02, *SE* = 0.11; S Table 7). We then estimated the across-gender genetic correlations for the two analyses and found that *r_g_* ranged from 0.88 (*SE* = 0.08) to 0.90 (*SE* = 0.08); these values were similar to those observed for the *AncestryDNA* specific analyses (Table 2; Table S7). Next, we tested whether the *AncestryDNA* lifespan associated loci were also significant in a meta-analysis of results from the UKB attained-age GWA.

We conducted the *AncestryDNA*-UKB meta-analysis of more than 658,000 individuals using *METAL* (Willer, Li, and Abecasis 2010). We included *AncestryDNA* GWA results from the broadly defined birth cohort (1886-1940; the larger of the two cohorts we investigated). Only variants that were common to both studies were included in the analysis; this requirement removed some variants that were found to be significant in the UKB study (details provided below).

**Figure 6:**
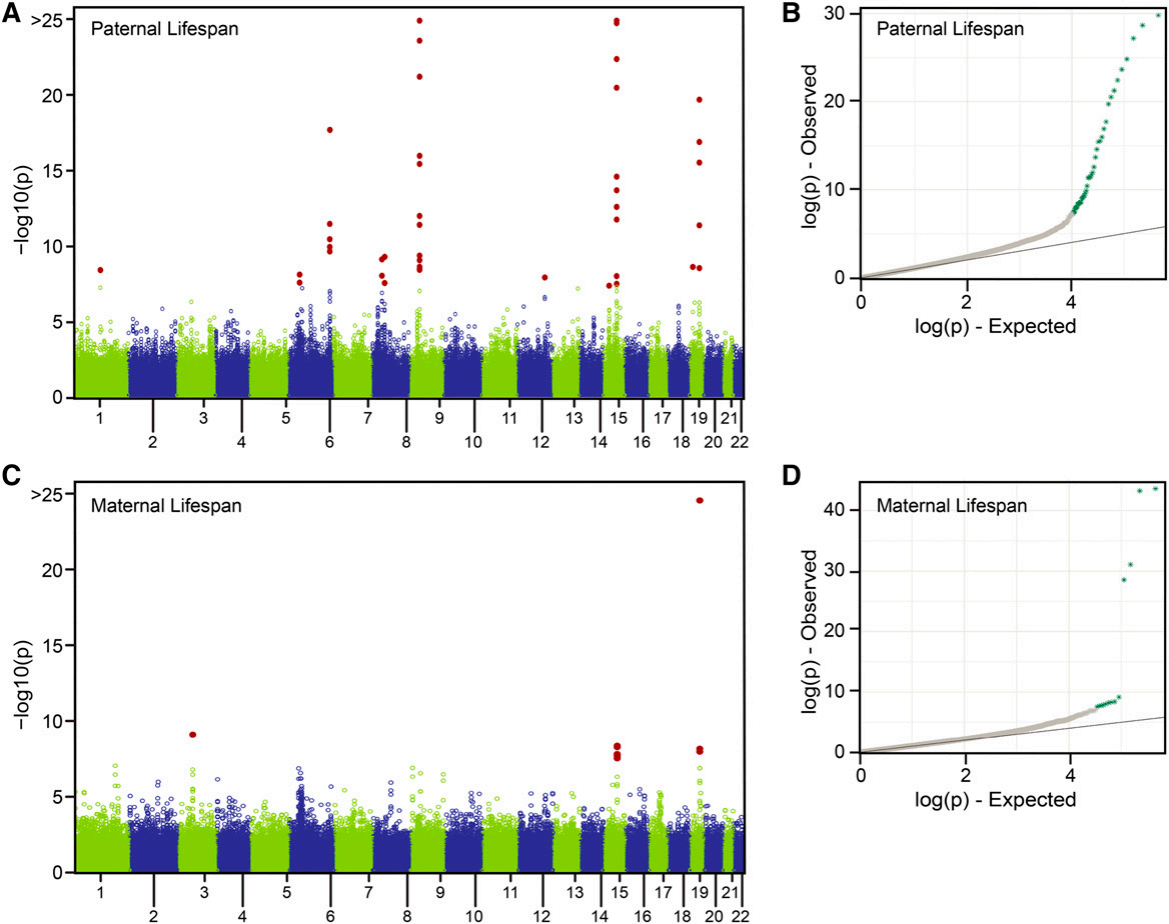
*AncestryDNA*-UKB meta-analysis results for maternal and paternal lifespan. Manhattan plots (A,C) where each circle shows the *P* value association test statistic for each genotyped SNP and solid red circles denote SNPs with Bonferroni corrected *P* values less than 0.05. QQ plots (B, D) of association test statistics, aquamarine colored points denote SNPs with Bonferroni corrected *P* values less than 0.05.

We found a total of 11 loci significantly associated with paternal lifespan (*P* < 5e-8) in the meta-analysis (Figure 6A,B). Of the five *AncestryDNA* paternal lifespan associated loci, three (*LPA*, *ANRIL*, and *CHRNA3/5*) maintained genome-wide significance in the meta-analysis, while *WAPL* and *SRRM3* failed to do so (Table 3). Of the 14 loci associated with attained-age of fathers in the UKB GWA (Pilling *et al.* 2017) genetic variants from ten loci were included in this meta-analysis (see Methods). Eight of those reached statistical significance (Table 3). In addition to the previously mentioned *LPA*, *ANRIL*, *CHRNA3/5* we found variants at: *APOE*, *CLESR2-PSRC1*, *ATXN2*, *SEMA6D*, and *MICA-MICB* were significant in the meta-analysis (Table 3). Two attained-age associated loci from the UKB analysis, *ZW10* and *C20orf187*, had *P* values greater than 5e-8 in the meta-analysis (Table 3). Finally, we identified significant associations at three loci, *LPL*, *EPHX2/CLU*, and *LDLR* (Table 3), which were marginally associated with paternal lifespan in the either the *AncestryDNA* or UKB studies.

**Table 3:** *AncestryDNA*-UKB GWA meta-analysis of parental lifespan. The *P* value is given of the lead variant at each locus in the test of association with parental lifespan in the *AncestryDNA*, UKB and combined cohorts. The *z*-score transformed variant effect sizes and standard errors are listed for the *AncestryDNA* and UKB analyses. Variants which were significantly associated with lifespan in either the *AncestryDNA* or UKB cohorts, and were not significant in the meta-analysis, are provided at the bottom of the table

					Meta-Analysis	*AncestryDNA*			UKB		
Trait	Locus	rsid	chr	bp	*P*	*P*	Effect (*z*-score)	*SE*	*P*	Effect (*z*-score)	*SE*
Paternal	*CLESR2..PSRC1*	rs599839	1	109822166	4.43e-09	1.66e-03	0.010	0.003	4.00e-09	0.015	0.003
Lifespan	*MICA..MICB*	rs3131621	6	31425499	8.86e-09	7.72e-04	−0.011	0.003	3.60e-08	−0.012	0.002
	*LPA*	rs140570886	6	161013013	1.13e-24	1.84e-13	−0.080	0.011	1.10e-17	−0.077	0.009
	*LPL*	rs15285	8	19824667	8.46e-10	2.44e-05	0.012	0.003	1.20e-07	0.013	0.002
	*EPHX2..CLU*	rs7844965	8	27442064	5.79e-10	9.06e-06	0.013	0.003	2.20e-07	0.013	0.003
	*ANRIL*	rs1556516	9	22100176	1.57e-28	2.99e-15	0.020	0.003	1.30e-20	0.020	0.002
	*ATXN2*	rs10774625	12	111910219	1.39e-08	9.97e-02	0.004	0.003	2.80e-12	0.015	0.002
	*SEMA6D*	rs4774495	15	47651362	4.92e-08	4.29e-03	−0.011	0.004	3.30e-08	−0.019	0.003
	*CHRNA3/5*	rs9788721	15	78802869	1.10e-31	8.49e-10	−0.016	0.003	1.40e-32	−0.027	0.002
	*LDLR*	rs6511720	19	11202306	2.73e-09	6.78e-05	0.016	0.004	1.70e-07	0.018	0.003
	*APOE*	rs769449	19	45410002	1.86e-20	4.31e-05	−0.016	0.004	5.60e-24	−0.033	0.003
Maternal	*IP6K1*	rs9872864	3	49792023	6.90e-10	5.61e-05	−0.011	0.003	1.30e-07	−0.011	0.002
Lifespan	*LPA*	rs186696265	6	161111700	8.43e-09	3.33e-03	−0.034	0.012	1.60e-08	−0.051	0.009
	*CHRNA3/5*	rs4887072	15	78925435	1.92e-09	3.82e-05	0.014	0.003	6.80e-07	0.013	0.003
	*APOE*	rs429358	19	45411941	4.37e-57	2.53e-09	−0.023	0.004	1.30e-68	−0.053	0.003
**Not Replicated**											
Paternal	*SRMM3*	rs28689051	7	75763624	3.41e-03	2.64e-09	0.016	0.003	2.60e-01	−0.003	0.002
Lifespan	*WAPL*	rs10887623	10	88315181	3.32e-03	3.36e-06	−0.020	0.004	9.20e-01	0.000	0.004
	*ZW10*	rs17613838	11	113638177	1.92e-04	8.11e-01	0.001	0.003	6.10e-09	−0.016	0.003
	*C20orf187*	rs6077996	20	10972839	2.28e-04	7.21e-01	0.001	0.003	4.60e-09	−0.013	0.002
Maternal Lifespan	*PSORS1C3*	rs1265159	6	31140047	6.43e-07	3.43e-01	−0.004	0.004	3.60e-10	−0.016	0.003

We found a total of four loci significantly associated with maternal lifespan in the meta-analysis, including *APOE*, the one locus significantly associated with maternal lifespan in the *AncestryDNA* dataset (Table 3; Figure 6C,D). Six loci were associated with maternal attained-age in the UKB GWA, and three of them, including *APOE*, were analyzed in the *AncestryDNA*-UKB meta-analysis. One locus, *LPA*, achieved statistical significance, whereas *PSORS1C3* failed to do so (Table 3). Finally, we identified two loci which, to our knowledge, have not been previously associated with maternal lifespan: *IP6K1* and *CHRNA3/5* (Table 3).

## Discussion

The *AncestryDNA*-UKB meta-analysis interrogated more than 658,000 samples and identified 11 loci significantly associated with paternal lifespan and four loci significantly associated with maternal lifespan. This analysis was the first large-scale, independent comparison of UKB parental lifespan GWA results.

### Many lifespan-associated loci were also linked with life-shortening diseases

We found variants at five loci (*ANRIL*, *LPA*, *APOE*, *LDLR*, *LPL*) that we refer to as candidate lifespan loci to emphasize that we identified a significant statistical association, while acknowledging that we have no functional-mechanistic data on how these genetic variants may affect lifespan. However, we suspect these loci to affect lifespan via increased risk for cardiovascular disease because they were previously associated with this disease (Nikpay *et al.* 2015; van der Harst and Verweij 2018) and it was the leading cause of death in the United States from 1980 to 2014 (Dwyer-Lindgren *et al.* 2016). *ANRIL*, *LPA* and *APOE* were significantly associated with parental lifespan in both the individual *AncestryDNA* and UKB analyses as well as the meta-analysis (Table 3). In contrast, *LDLR* and *LPL* were only significantly associated with lifespan in the large meta-analysis and marginally associated with lifespan in the individual *AncestryDNA* and UKB analyses (Table 3).

The *CHRN3/5* locus has been linked to smoking behavior and incidence of chronic obstructive pulmonary disease (Hobbs *et al.* 2017), another leading life-threatening disease in the United States (Dwyer-Lindgren *et al.* 2016). This locus was significantly associated with paternal lifespan in both the *AncestryDNA* and UKB independent analyses. In the meta-analysis, the combined power of both cohorts also revealed this locus to also be significantly associated with maternal lifespan (Table 3).

We found two lifespan candidate loci which were previously associated with Alzheimer’s disease (Lambert *et al.* 2013): the newly identified *EPHX2/CLU* and, perhaps the most widely reported candidate lifespan locus: *APOE* (Deelen *et al.* 2011; Deelen *et al.* 2014; Broer *et al.* 2015). Cohort-specific analysis of *APOE* revealed two curious results: 1) *APOE* exhibited a negative effect on lifespan in older cohorts and a positive effect in younger cohorts (Figure 5E) and 2) the minor allele frequency increased from 11 to 15% between the 1886 and 1940 birth cohorts (Figure 5G). The minor allele at *APOE* was at highest frequency for intermediate lifespan values (74-86 years). This pattern was most pronounced in the younger birth cohorts, and it suggested that this allele (or a linked allele or alleles) confers a survival benefit early in life but a survival detriment later in life. While it may be tempting to invoke the antagonistic pleiotropy theory of aging to explain such a result (Williams 1957), two variants in tight linkage disequilibrium, which could even modify the action of two different genes in this gene-dense region (S Figure 8), would equally explain this result.

Finally, three loci had no known disease associations. *WAPL* and *SRRM3*, identified in the *AncestryDNA* analysis (Table 1) and *IP6K1*, identified in the meta-analysis (Table 3) have, to our knowledge, not been associated with any life-shortening diseases.

### Heterogeneity of GWA results across analyses

Several loci that were significantly associated with lifespan in either the *AncestryDNA* analysis (*WAPL* and *SRRM3*) or the UKB analysis (maternal lifespan: *PSORS1C3*; paternal lifespan: *ZW10* and *C20orf187*) did not achieve significance in the meta-analysis. One explanation is that these loci do not affect parental lifespan. This could be due to underlying population structure that was not fully accounted for in the GWA mapping models. If so, the signal of association would not be replicated in the comparison between the primarily-British *vs.* primarily-American cohorts. Alternatively, the candidate loci may directly affect parental lifespan, but distinct population structure, environmental hazards, or phenotypic definitions between the two studies may have resulted in a failure to replicate across independent cohorts.

A second factor to consider is that the focal trait examined in our *AncestryDNA* study was lifespan and only included deceased parents, whereas the UKB study examined the attained-age of both living and deceased parents. We reiterate that we did not include parents lacking a death date in the *AncestryDNA* analysis because we could not determine whether any specific individual was in fact alive or deceased and their date of death was never recorded (Ruby *et al.* 2018, Figure 1G). The difference between focal traits implies that the UKB population included younger individuals from more recent birth cohorts compared to *AncestryDNA*, and differences in the environment or population sub-structure between historical eras may have contributed to the lack a replication between studies.

### Differences in the genetic architecture of lifespan *vs.* age

The difficulty of obtaining genetic information from deceased individuals has motivated investigators to use age as a proxy phenotype for human lifespan (Schächter *et al.* 1994; Willcox *et al.* 2008; Newman *et al.* 2010; Soerensen *et al.* 2010; Deelen *et al.* 2011; Nebel *et al.* 2011; Beekman *et al.* 2013; Soerensen *et al.* 2013; Sebastiani *et al.* 2013; Deelen *et al.* 2014; Broer *et al.* 2015; Sebastiani *et al.* 2017). We applied that strategy in this study. However, we also addressed this issue using a second strategy: we examined the complete lifespans of parents and the genotypes of their offspring (similar to the approach formalized in Liu *et al.* (2017)). The genetic correlations between the outcomes of these two strategies was relatively high, ranging from 0.43 to 0.70 in gender specific comparisons (Table 2). Nonetheless, of the several loci that were associated with either age or parental lifespan to a statistically significant degree, only the *APOE* locus was associated with both (Figure 4I; Figure S10). These results suggested that the use age as a proxy for lifespan in a GWA analysis is informative but not comprehensive.

The differences in GWA mapping results between age and lifespan were likely due, at least in part, to the fundamental nature of the two traits. Lifespan is a property of deceased individuals and was measured prospectively on individuals from the same birth cohort. Age is a property of the living and GWA analyses of this trait are retrospective comparisons between differentially aged individuals from distinct birth cohorts. Prospective GWA analysis of lifespan potentially avoids problems that are specific to retrospective studies: recruitment bias of long-lived individuals and establishment of an appropriate youthful control population (Clayton and McKeigue 2001; Manolio, Bailey-Wilson, and Collins 2006). Recruitment biases may explain why the association between *APOE* and current age is much stronger than with lifespan; variants at this locus significantly increases the incidence of Alzheimer’s disease in old age, and may inhibit participation in web-based genealogy services and in providing informed consent to research.

### Little heritability is “missing” from the SAP cohort

We estimated *h_g_^2^* for paternal and maternal lifespan to be between 2 and 8%, depending on the cohort examined (Figure 3F, 4B). For many phenotypes, it has been noted that estimated *h_g_^2^* values, measured using GWA methods, are generally much less than *h^2^* values measured based on familial phenotypic correlations; this recurring discrepancy is known as the “missing heritability” problem (Manolio *et al.* 2009). While our *h_g_^2^* values could potentially have been increased through the evaluation of genome-wide imputed variants (Yang *et al.* 2010; Yang *et al.* 2015), our previously published measure of lifespan heritability derived from familial correlations in the same SAP used for this analysis (Ruby *et al.* 2018) produced a range of *h^2^* values, all below 10%. While our estimate of *h_g_^2^* was slightly lower than our estimate of *h^2^*, the values reported in each study cover the same range, giving the impression that there was little heritability remaining to be explained.

In comparison to previously published estimates of *h^2^* for human lifespan, our estimate of *h_g_^2^* was low, and would have been interpreted as consistent with the “missing heritability” problem (Manolio *et al.* 2009). For instance, another large-pedigree study recently estimated *h^2^* to be between 16 and 18% (Kaplanis *et al.* 2018), and multiple smaller studies estimated *h^2^* to be in the range of 15–30% (Philippe 1978; Mayer 1991; Herskind *et al.* 1996; Ljungquist *et al.* 1998; Gavrilov *et al.* 1998; Mitchell *et al.* 2001; Kerber *et al.* 2001; Gögele *et al.* 2011). Had those estimates of heritability been used, the apparently “missing” component following our present analysis would have been quite large. Importantly, our own estimates of under 10% overall heritability for lifespan differed from the precedent literature not by the familial correlations, but rather by our accounting for considerable assortative mating (Ruby *et al.* 2018). Therefore, our observation that the small difference between *h^2^* and *h_g_^2^* is due to the consideration of assortative mating is an alternative hypothesis to the often invoked explanations: rare variants, structural variation, or epistatic interactions (Manolio *et al.* 2009). The extent of assortative mating, either by primary or secondary means, has not been thoroughly explored for many other phenotypes. It is worth considering that the inflation of *h^2^* estimates due to assortative mating may be more generally responsible for the recurrence of sizable “missing heritability” gaps.
